# Inhibition of Complement Retards Ankylosing Spondylitis Progression

**DOI:** 10.1038/srep34643

**Published:** 2016-10-04

**Authors:** Chaoqun Yang, Peipei Ding, Qingkai Wang, Long Zhang, Xin Zhang, Jianquan Zhao, Enjie Xu, Na Wang, Jianfeng Chen, Guang Yang, Weiguo Hu, Xuhui Zhou

**Affiliations:** 1 Department of Spine Surgery, Changzheng Hospital, Second Military Medical University, Shanghai 200003, China; 2 Department of Hand Surgery, Huashan Hospital, Fudan University, 12 Wulumuqi Middle Road, Shanghai, 200040, China; 3 Fudan University Shanghai Cancer Center and Institutes of Biomedical Sciences, Collaborative Innovation Center of Cancer Medicine, Department of Oncology, Shanghai Medical College, Fudan University, Shanghai 200032, China; 4 Beijing Institute of Basic Medical Sciences, 27 Taiping Road, Beijing 100850, China; 5 Department of Immunology, Shanghai Medical College, Fudan University, Shanghai 200032, China

## Abstract

Ankylosing spondylitis (AS) is a chronic axial spondyloarthritis (SpA) resulting in back pain and progressive spinal ankyloses. Currently, there are no effective therapeutics targeting AS largely due to elusive pathogenesis mechanisms, even as potential candidates such as HLA-B27 autoantigen have been identified. Herein, we employed a proteoglycan (PG)-induced AS mouse model together with clinical specimens, and found that the complement system was substantially activated in the spinal bone marrow, accompanied by a remarkable proportion alteration of neutrophils and macrophage in bone marrow and spleen, and by the significant increase of TGF-β1 in serum. The combined treatment with a bacteria-derived complement inhibitor Efb-C (C-terminal of extracellular fibrinogen-binding protein of *Staphylococcus aureus*) remarkably retarded the progression of mouse AS by reducing osteoblast differentiation. Furthermore, we demonstrated that two important modulators involved in AS disease, TGF-β1 and RANKL, were elevated upon *in vitro* complement attack in osteoblast and/or osteoclast cells. These findings further unravel that complement activation is closely related with the pathogenesis of AS, and suggest that complement inhibition may hold great potential for AS therapy.

Ankylosing spondylitis (AS) is a chronic, progressive inflammatory autoimmune disease mainly afflicting the sacroiliac joints and spine, and is considered to be a “prototype” of spondyloarthritis (SpA). The major clinical features of AS include back pain and progressive stiffness of the spine^1^,^2^. The strong association between self-recognized human leukocyte antigen B27 (HLA-B27) and susceptibility to AS has been proposed over the past three decades; however, most individuals who test positive for HLA-B27 are healthy, thus the pathogenic mechanism underlying this association remains unclear. In addition, other putative autoantigens have been implicated in the etiology of AS, including *Klebsiella* antigens^3^, *Yersinia* antigens^4^. and epitopes in the cartilage PG^5^. Unfortunately, there are no effective disease-modifying treatment strategies for AS currently. Continuous administration of non-steroidal anti-inflammatory drugs (NSAIDs) are still the first-line treatment for AS, which show a significant reduction in radiographic progression despite ongoing inflammatory pain symptoms and disease activity^6^. For patients with inadequate response to NSAIDs, the second line of treatment are tumor necrosis factor-alpha (TNF-α) inhibitors. These TNF-α inhibitors have resulted in improved symptoms and functionality in approximately 60% of AS patients^7^,^8^,^9^,^10^. However, anti-TNF-α treatment has not shown improvement in radiographic progression^11^,^12^,^13^ unless treatment is initiated at the early disease stage and with longer duration of follow-up^14^. In addition, other biologic agents, including abatacept that targets CTLA-4, tocilizumab and sarilumab targeting IL-6R and anakinra that targets IL-1, showed minimal efficacy in AS despite their efficacy in other inflammatory rheumatic diseases^15^. Therefore, further research on the pathogenesis of AS is necessitated for developing more effective intervention of this disorder.

The complement system is a central effector of innate immunity whose functions extend from eliminating foreign pathogens to orchestrating immune responses and contributing to homeostasis mainly *via* its cleaved products, including the key pro-inflammatory C3a and C5a, opsono-cytophagic C3b/iC3b, and cytolytic membrane attack complex (MAC, comprising C5b-9n components)^16^. However, complement dysfunctions, including uncontrolled activation and insufficient regulation, turns its destructive capabilities against host cells, suggesting the complement system is an important contributor to various human diseases, such as autoimmune, inflammatory, and infectious diseases^17^. Numerous studies demonstrated complement activation in AS by the significantly elevated complement components or activation products including C3, C4 and C3d, and by the complement activation triggers including IgA, IgG, C-reactive protein (CRP), serum amyloid A, apolipoprotein A^18^,^19^,^20^,^21^,^22^,^23^,^24^. Among these triggers, the cross-reactive antibodies against autoantigens such as *Klebsiella*, when present in high titers, may activate the classical complement cascade^3^. However, the influence of complement inhibition in AS has not been elucidated yet.

In this study, we found that the complement system was substantially activated in PG-induced AS mice and/or clinical specimens accompanied by the up-regulated TGF-β1 serum levels. A bacteria-derived complement inhibitor Efb-C remarkably retarded the AS progression in our mouse model. *In vitro* experiments also demonstrated that complement activation could increase the levels of TGF-β1 and/or RANKL in osteoblasts or osteoclasts. Our findings establish a proof of concept that complement inhibition holds great potential for AS therapeutics.

## Results

### Complement inhibitor Efb-C markedly retards the disease progression in PG-induced AS mouse model

To determine the effect of complement inhibition on the progression of AS, we first prepared a bacteria-derived complement inhibitor, recombinant 6 × His tagged Efb-C (11.9 kDa). Efb-C could intercept complement cascade by specifically binding to C3/C3b, which was truncated from C-terminal of intact Efb according to the previous report^25^. As shown in Supplementary Figure S1A, B, the recombinant Efb-C with high purity could effectively block classical complement activation with IC_50_ of 121 μg/ml.

**Figure 1:**
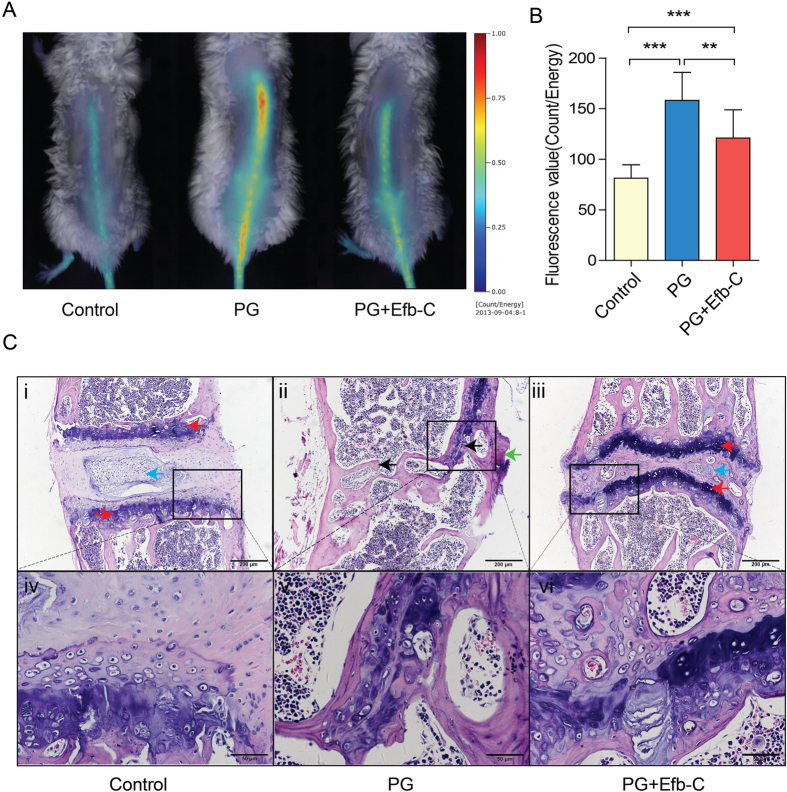
Efb-C alleviated the progression of AS in mouse model. (**A**) Efb-C reduced osteoblast activity *in vivo* assessed by OsteoSense 750 EX probe. (**B**) Fluorescence values were quantitatively scored. Data represent mean ± SEM; Control, n = 10; PG, n = 10; PG + Efb-C, n = 9; **P < 0.01, ***P < 0.001. (**C**) The representative thoracic Spine segments were stained with H&E from control (i, iv), PG alone (ii, v), and PG + Efb-C (iii, vi) treated mice. Control group mice showed the normal intervertebral joint (i and iv), whereas PG alone treatment resulted in the severe destruction of intervertebral joint determined by the disappeared intervertebral space, cartilage (red arrow) and vertebral nucleus pulposus (blue arrow), the formation of excessive bone matrix and syndesmophyte (green arrow), and the joint fusion (black arrow) accompanied by the formation of newborn marrow cavity with the embedded hematopoietic cells (ii and v), which were remarkably alleviated by the additional Efb-C treatment (iii and vi). Scale bars, 200 μm in images i–iii and 50 μm in images iv–vi.

Next, we determined whether Efb-C could impede the disease course in PG-induced AS mouse model through complement inhibition. AS is initiated by erosion and more importantly, primarily characterized by osteoproliferation and consequent ankyloses with high osteoblast activity^1^. OsteoSense 750 EX probe labeled with near infrared (NIR) fluorescent dyes can bind to newly synthesized hydroxyapatite by osteoblasts, thus fluorescent intensity indicates osteoblast activity and the resulting microcalcifications and bone remodeling which can further represent the severity of AS. Herein, mice treated with PG alone displayed significantly stronger fluorescent intensity thus indicative of increased osteoblast activity than control mice; and PG + Efb-C treatment decreased osteoblast activity represented by the lower fluorescent intensity than PG alone treatment (Fig. 1A), which was further determined by the result of quantitative analysis (Fig. 1B). These data suggest that complement inhibition may markedly, though not completely, retard the progression of PG-induced AS in mouse model (Fig. 1A,B). Although the low immunogenicity of Efb-C benefits pharmacologic effect (see Supplementary Figure S1C), which also excluded the possible effect of immune response and subsequent complement activation induced by the potential antibody against Efb-C, these findings strongly suggest the urgent requirement for development of therapeutic complement inhibitors in AS treatment.

At the endpoint of the experiment, we further collected the mouse spines for histological analysis. In control mice, the H&E staining showed intact joints, in which the intervertebral space physiologically existed with the regular intervertebral cartilage and epiphyseal plate although the vertebral nucleus pulposus was slightly calcified due to old age (images i and iv in Fig. 1C). However, in PG-treated mice, we observed that the represented intervertebral discs (IVDs) in thoracic spine were severely destructed. The normal intervertebral space, cartilage and vertebral nucleus pulposus completely disappeared, leading to excessive bone matrix and syndesmophyte formation, and eventual joint fusion followed by the formation of newborn marrow cavity with the embedded hematopoietic cells (images ii and v in Fig. 1C). Upon Efb-C administration to PG-treated mice, we observed a remarkably ameliorated degree of IVDs destruction. We did not find any osteo-ankylosis by H&E staining in all detected mice with Efb-C treatment although the inflammatory joint damage was worse than control mice. The intervertebral space and cartilage were still retained though with slight anatomical disorder. The vertebral nucleus pulposus was slightly calcified accompanied by embedded marginal hematopoietic cells, whereas the epiphyseal plate still clearly reserved (images iii and vi in Fig. 1C). Therefore, these results implicate that complement inhibition noticeably retarded the AS progression in a PG-induced mouse model.

### Efb-C Inhibited PG-induced Complement Activation on Sites of Vertebral Bone Marrow

**Figure 2:**
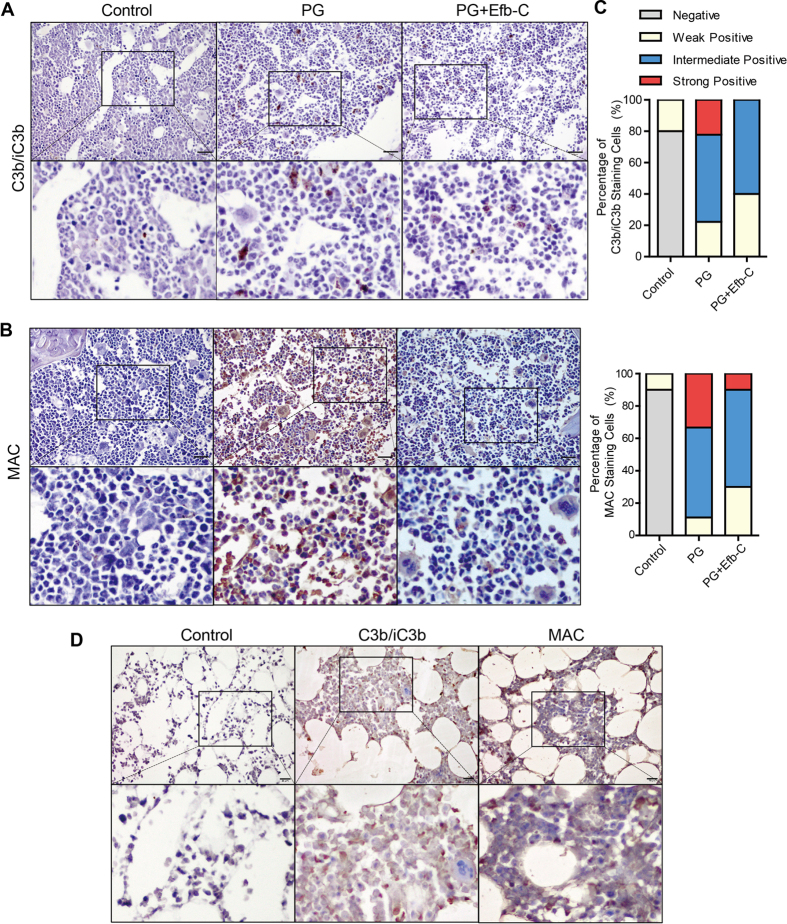
Complement was extensively activated by PG immunization and intercepted by additional Efb-C treatment in the spinal bone marrow in a PG-induced AS mouse model and in AS patients detected by IHC analysis. (**A,B**) PG immunization induced complement cleavage products C3b/iC3b exclusively deposited in macrophages (**A**) and MAC/C5b-9 deposited in various types of cells (**B**) compared with control treatment, which was evidently alleviated by the additional Efb-C treatment. See also Supplementary Figure S2. (**C**) Quantitative data of immunohistochemical analysis in (**A**,**B**). (**D**) The extensive complement activation was determined by C3b/iC3b and MAC deposition in most types of cells in bone marrow of facet joints collected from an ankylosing spondylitis patient with correction surgery. Lower panel shows a 10× magnification of the area outlined by the black rectangle in the upper image. Scale bars, 20 μm.

Since the PG for inducing mouse AS was prepared from human articular cartilage, we detected the initiated sites of complement activation by the mouse anti-human PG antibodies at the periphery of the IVDs. To investigate the status of complement activation in mice with different treatment using PBS, PG alone or PG combined with Efb-C, mouse spines were collected and stained with antibodies against C3b/iC3b or MAC in IHC assay. We found that C3b/iC3b sporadically deposited in a certain type of cells in spinal bone marrow of PG-treated mice (Fig. 2A), which was further identified to be macrophages by the staining with C3b/iC3b or F4/80 in two serial sections (see Supplementary Figure S2). Efb-C treatment resulted in fewer and weaker staining of C3b/iC3b than PG alone treatment (Fig. 2A). However, nearly all types of cells were strongly stained by MAC antibody in PG alone treated mice, whereas the combined treatment with Efb-C markedly reduced the amount of positively-stained cells and intensity of staining (Fig. 2B). In addition, we did not observe the specific C3b/iC3b or MAC stainings in control mice (Fig. 2A,B), indicating the specificity of immunostaining of C3b/iC3b and MAC. The quantitative analysis data shown in Fig. 2C further support the above finding that complement cascade is extensively activated in PG-immunized mice which can be attenuated by Efb-C administration.

We further detected the complement activation by C3b/iC3b and MAC staining in vertebrae facet joint tissues of two AS patients underwent orthopedic surgery. We observed that the extensively positive-staining of both C3b/iC3b and MAC (Fig. 2D), in which C3b/iC3b deposition in nearly all cell types was different from the exclusive expression in macrophages in PG-induced AS mice, implicating more types of cells were targeted by the complement system in AS patients.

### Efb-C Treatment Ameliorated PG-induced Inflammation

**Figure 3:**
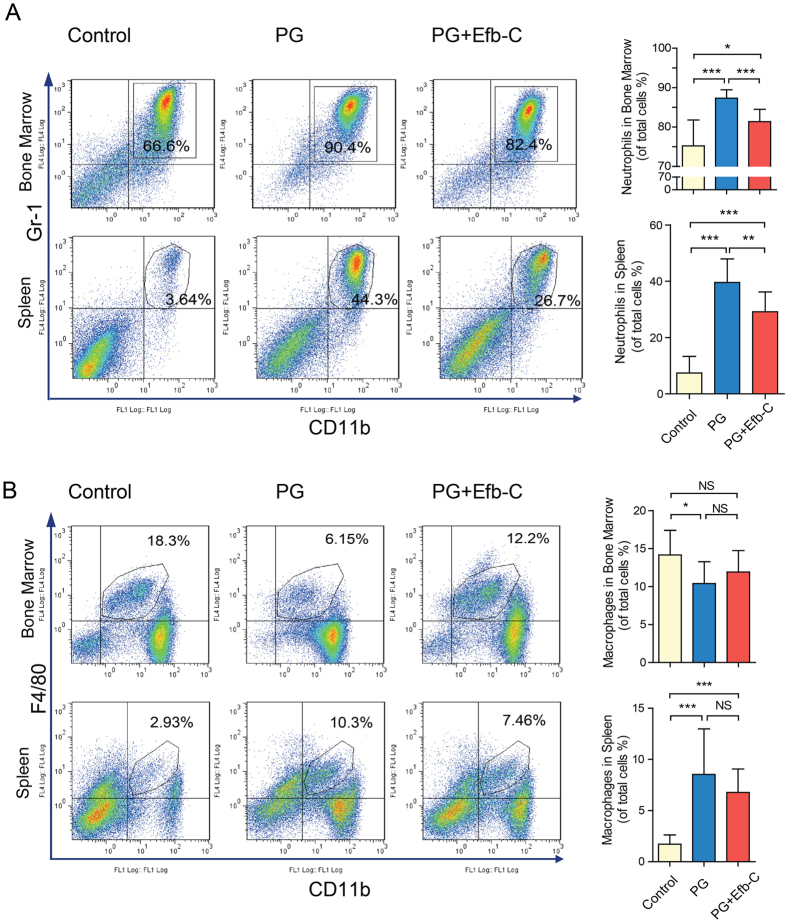
PG immunization induced the proliferation or differentiation of inflammatory cells that could be attenuated by additional Efb-C treatment in AS model. (**A**) PG immunization enhanced the proliferation of neutrophil (Gr-1+, CD11b+) in bone marrow and spleen compared with control treatment, which could be attenuated by the additional Efb-C treatment. (**B**) Macrophages (F4/80+, CD11b+) reduced in bone marrow of PG-immunized mice likely due to its differentiation into multinucleated osteoclasts compared with control treatment, which was attenuated by the additional Efb-C treatment. However, more macrophages were observed in spleen of PG-immunized mice compared with control treatment, which was reduced by Efb-C treatment to a certain degree. Right figures represent the quantitative data. Control, n = 10; PG, n = 8; PG + Efb-C, n = 9.

To assess whether PG immunization would stimulate the complicated cellular and molecular inflammatory response in our AS mouse model, we first measured the alteration of neutrophils (Gr-1 + CD11b+) and macrophages (F4/80 + CD11b+) in bone marrow and spleen, two major contributor cells in inflammation. We observed that neutrophils increased significantly both in the bone marrow and spleens of PG-treated mice than control mice, which could be markedly reduced *via* Efb-C treatment, although neutrophils in Efb-C treatment group were still significantly higher than those in the control group (Fig. 3A). In contrast, unlike neutrophils, bone marrow macrophages, i.e., osteoclasts precursors significantly decreased due to PG immunization as compared to control treatment, which was partially restored by Efb-C treatment though no statistical significant difference was observed (Fig. 3B). However, more macrophages were detected in spleen of PG alone and PG + Efb-C treated groups than in control mice, in which the added Efb-C treatment reduced the amount of macrophages compared with PG alone treatment although there was no significance between them (Fig. 3B). Therefore, these results indicate that PG immunization strongly induced the proliferation and differentiation of immune cells, in which the macrophages probably differentiated into osteoclasts. More importantly, treatment with Efb-C could obviously prevent this process.

**Figure 4:**
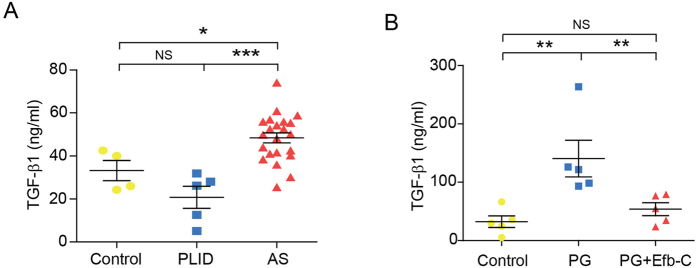
Elevated TGF-β1 serum levels in AS patients detected by a commercial ELISA kit. (**A**) and in mouse model detected by a BioPlex assay (**B**). PLID, Prolapse of Lumbar Intervertebral Disc; AS, Ankylosing Spondylitis. Healthy control, n = 4; PLID, n = 5; and AS, n = 22 (**A**). N = 5 mice in each group (**B**). Data represent mean ± SEM. NS, no significance; *P < 0.05; **P < 0.01; and ***P < 0.001. See also Supplementary Figure S3.

Furthermore, mouse sera were collected and employed to measure the concentrations of a panel of cytokines with Bio-Plex system. Serum TGF-β1 level has been reported to be significantly elevated in AS patients^26^, thus playing a critical role in both osteoblastogenesis and osteoclastogenesis which was extensively discussed in an elegant review^27^. Indeed, we found that serum TGF-β1 level in AS but not PLID (Prolapse of Lumbar Intervertebral Disc) patients was remarkably elevated than that in healthy controls (Fig. 4A). Consistent with human data, the serum concentration of TGF-β1 in PG-immunized mice was higher than that in either control or Efb-C-treated mice, and the elevated TGF-β1 level decreased obviously *via* Efb-C treatment to a comparable level in control mice (Fig. 4B). In addition, serum MIP-1α levels has been reported to be higher in AS patients than those in rheumatoid arthritis (RA) patients and healthy controls, and anti-TNFα therapy appeared to reduce serum MIP-1α levels though with lower efficacy in AS than in RA patients^28^. Interestingly, serum MIP-1α levels displayed the similar pattern with TGF-1β in PG-induced AS mouse model, i.e., it significantly increased in PG immunized mice as compared to control and Efb-C-treated mice, which was reduced by Efb-C treatment though without statistical significance (see Supplementary Figure S3, bottom left panel). However, the other 27 tested cytokines (IL-1α, IL-1β, IL-2, IL-3, IL-4, IL-5, IL-6, IL-10, IL-12(p40), IL-12(p70), IL-13, IL-17, Eotaxin, G-CSF, GM-CSF, IFN-γ, KC, MCP-1, VEGF, MIP-1β, RANTES, TNF-α, IL-18, FGF-basic, M-CSF, and TGF-β3) except for TGF-β2 did not show any meaningful pattern among three groups of mice (see Supplementary Figure S3).

### Complement Activation Enhanced Osteoblast and Osteoclast Activity *in vitro*

**Figure 5:**
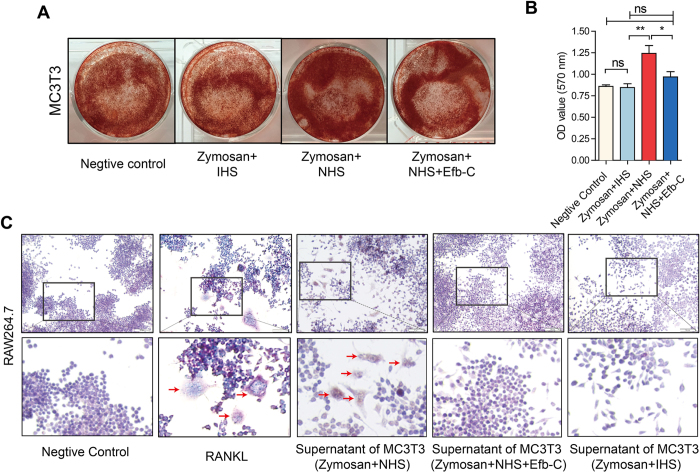
Complement Activation Enhanced Osteoblastogenesis and Osteoclastic Differentiation *in vitro* Alizarin Red S staining: Complement activation by zymosan and NHS treatment could result in the osteoblastogenesis of MC3T3-E1 cells. (**B**) Statistical analysis of mineral deposition in (**A**). Data are presented as the mean ± SEM. The significant differences between two groups were determined using the one-tailed Student’s *t* test for unpaired data. ns, no significance; *P < 0.05; and **P < 0.01. (**C**) TRAP staining: both RANKL (50 ng/ml) and culture supernatant of MC3T3-E1 cells sustained complement attack by zymosan and NHS could induce the osteoclastic differentiation of RAW264.7 cells. Images are representative of three independent experiments.

MC3T3-E1 and RAW 264.7 are murine osteoblast precursor and monocytic cell, respectively, which were used to investigate the effect of complement activation on the activation of osteoblast and osteoclast. In functional test we observed that complement activation in MC3T3-E1 cells treated by normal human serum (NHS) and zymosan obviously induced osteoblastogenesis visualized by Alizarin Red S staining and quantitative analysis as compared to control, NHS and zymosan together with Efb-C, or heat-inactivated human serum (IHS) combined with zymosan treatment (Fig. 5A,B). Furthermore, we employed the cell culture supernatant of MC3T3-E1 treated by NHS and zymosan to detect the osteoclastic differentiation of RAW264.7 cells by TRAP staining, and found a notable cell differentiation in a comparable level with the positive control of RNAKL treatment. However, the osteoclastic differentiation did not appear in the negative control, or treatment with IHS and zymosan or with NHS, zymosan and Efb-C (Fig. 5C).

**Figure 6:**
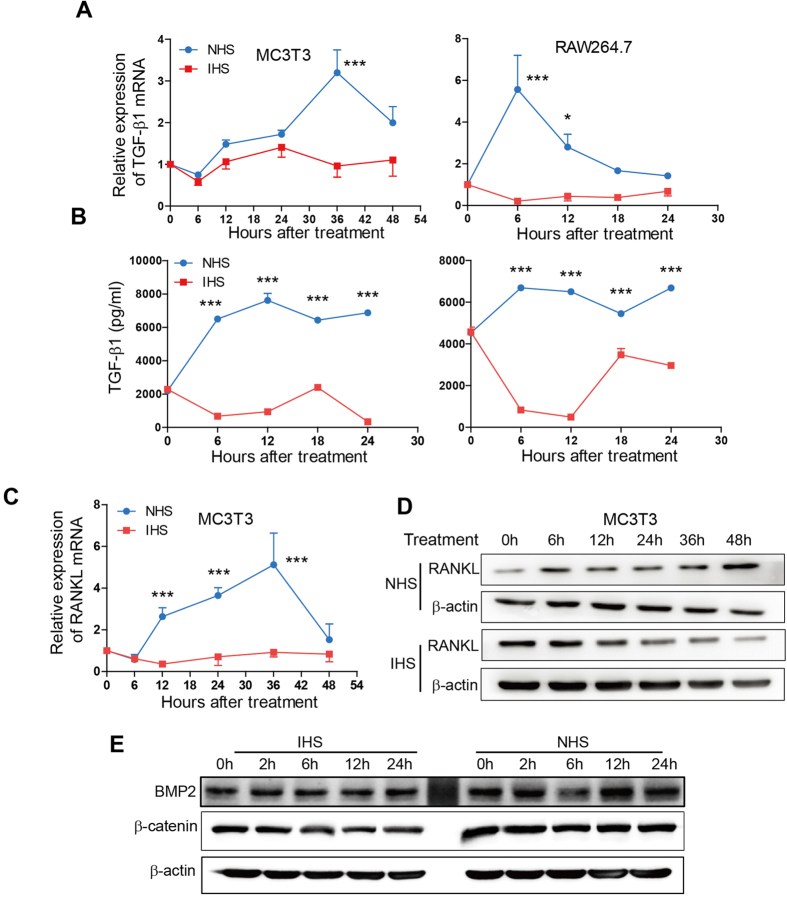
Complement Activation Increase the levels of TGF-β1 and/or RANKL. (**A**–**D**) Complement activation by zymosan and NHS treatment (blue curve) increased the levels of *TGF-β1* mRNA (**A**) and protein (**B**) in MC3T3-E1 and RAW264.7 cells, or the levels of *RANKL* mRNA (**C**) and protein (**D**) detected by qRT-PCR (**A**,**C**), ELISA (**B**) or immunoblotting (**D**) compared with zymosan and IHS treatment (red curve). Experiment was performed in triplicate and data represents mean ± SEM. *P < 0.05; **P < 0.01; and ***P < 0.001. (**E**) Complement activation slightly increased the level of BMP2 but not β-catenin. The blot images shown in (**D**) and (**E**) were cropped, and the original images correspond to Figure S4A,B, respectively.

We further investigated the mechanisms for complement activation increasing the activities of osteoblast and osteoclast. As shown in Fig. 6A, complement activation by zymosan combined with NHS treatment remarkably increased the *TGFB1* transcription at 36 hours in MC3T3-E1 cells or at 6 and 12 hours in RAW264.7 cells compared with IHS treatment. In agreement, TGF-β1 protein concentration in cell culture supernatant also increased in both cell lines with NHS treatment (Fig. 6B). We observed that this increase of TGF-β1 protein levels starting from 6 hours occurred earlier (in MC3T3 cells) or similar with (in RAW264.7 cells) the increase of mRNA levels, implicating that there may be other mechanisms such as effect of MAC pore than transcription regulation for the up-regulated TGF-β1 protein level provoked by complement activation. Numerous reports demonstrated that the MAC pore could constitute a transient route to allow for the export of cytosolic proteins such as bFGF, IL-1β, IFN-γ^29^,^30^,^31^,^32^. Meanwhile, we observed that complement activation by NHS treatment in osteoblastic MC3T3-E1 cells significantly enhanced the transcription of *RANKL* starting from 12 to 36 hours as comparison to IHS treatment (Fig. 6C). Immunoblotting assay further demonstrated that the protein level of RANKL was upregulated only by NHS treatment starting from 6 hours (Fig. 6D). In addition, using western blot assay we also detected the potential effect of complement activation on expressions of another TGF-β superfamily member BMP-2 and β–catenin in MC3T3-E1 cells. However, only the level of BMP-2 was slightly increased at 12 hours after complement challenge (Fig. 6E). Therefore, the effect of complement activation on osteoclastic differentiation of RAW264.7 cells most likely results from the proteins such as TGF-β1 and RANKL secreted from MC3T3-E1 cells stimulated by complement activation.

## Discussion

Consistent with the previous concept that the complement system is activated in AS disease^3^,^18^,^19^,^20^,^21^,^22^,^23^,^24^, in this study we found that two representative products of complement activation, C3b/iC3b and MAC were comprehensively deposited on many bone marrow cells at the periphery of the IVDs in the PG-induced AS mouse model and clinical AS specimen (Fig. 2, and Supplementary Figure S2). Furthermore, PG immunization promoted the intense proliferation and/or differentiation of neutrophils and macrophages in bone marrow and enhanced peripheral distribution such as in spleen (Fig. 3). Of note, the proportion of macrophages in bone marrow of PG alone treated mice exclusively decreased as compared to control treatment. Macrophages, i.e., osteoclasts precursors can be positively stained by anti-F4/80 antibody, whereas osteoclasts have been report to lack F4/80 expression^33^. Therefore, this phenomenon most likely resulted from the differentiation of the expected more macrophages in PG-treated mice into osteoclasts than in control mice. Meanwhile, the serum level of TGF-β1, an important molecule modulator in AS significantly was determined to increases in PG-induced AS mouse model and in AS patients (Fig. 4), which was recapitulated by the complement attack on both osteoblast and osteoclast *in vitro* (Fig. 5). Therefore, these findings logically implicate that complement activation likely play an important role in the pathogenesis of AS and complement inhibition may retard AS progression. Indeed, treatment with the complement inhibitor Efb-C markedly, though not completely, impeded the above inflammatory response and retarded the AS progression in a PG-induced AS mouse model (Figs 1, 3 and 4B). This partial achievement was probably attributable to the incomplete complement inhibition by Efb-C due to its high IC_50_ value (121 μg/ml) and poor pharmacokinetic/pharmacodynamic (PK/PD) features resulting from its low molecular weight. Therefore, these results suggest the strong needs for development of therapeutic complement inhibitors in AS treatment.

The pathogenesis of AS is involved with a bone remodeling cycle consisting of bone resorption, removal and formation induced by hyperactive osteoblast and osteoclast activity^34^. The osteoblast precursors may be activated by systemic or local regulators such as parathyroid hormone (PTH), parathyroid hormone-like hormone (PTHLH), calcitriol, insulin-like growth factors (IGFs), bone morphogenetic proteins (BMPs) and TGF-β1. The activated osteoblast precursors initiate this cycle, further express receptor activator of NF-kappa B ligand (RANKL) that plays a central role in osteoclastogenesis^35^. RANKL interplays with RANK, a receptor on osteoclast precursors, thus resulting in activation, differentiation, and fusion of osteoclast precursors for entering into the process of resorption^34^. During this cycle, TGF-β1, the most abundant and important factor in bone environment, plays a major role in bone development and maintenance, and triggers a positive feedback by affecting both osteoblasts and osteoclasts^27^. On one side, the up-regulated TGF-β1 that is induced by the osteoclast activation through RANKL/RANK interaction is subsequently able to activate osteoblasts. On the other hand, TGF-β1 can directly stimulate osteoclast precursors differentiation and enhance osteoclastogenesis^36^ by increasing RANK expression at both mRNA and protein levels^37^ through interaction between Smad3 and Traf6 in the presence of RANKL and M-CSF^38^. Therefore, TGF-β1 and RANKL are two critical modulators for regulating the activities of osteoblasts and osteoclasts in AS disease progression. In this study, we demonstrated that TGF-β1 serum level was increased in a PG-induced AS model and in AS patients and, more importantly, both of TGF-β1 and RANKL in osteocytes were up-regulated upon complement attack *in vitro*, which strongly implicates the involvement of complement activation in the pathogenesis of AS.

The majority of complement components are normally biosynthesized in liver and further secreted into circulation for their function; however, an *in vitro* experiment discovered the complement resource and triggers of complement activation^39^. The complement components can be locally produced by mesenchymal stem cells, osteoblasts and osteoclasts then activated by osteoblasts and particularly osteoclasts. Furthermore, C3a and C5a not only, with the co-stimulation of IL-1β, significantly up-regulated the expression of inflammatory cytokines IL-6 and IL-8 as well as the expression of RANKL and osteoprotegerin (OPG) from osteoblasts, but also directly induced the process of osteoclastogenesis but not osteogenic differentiation^39^. In addition, C3a has recently been reported to induce TGF-β1 expression in normal primary human small airway epithelial cells^40^. Furthermore, there is possible crosstalk between TGF-β1 signaling and complement activation. TGF-β1 down-regulates complement inhibitory proteins CD46 and CD55 expression *via* the p38MAPK/Snail axis to enhance complement activation, thus producing high levels of C3a and C5a that can suppress SMAD7 effectively^40^. Therefore, this positive feedback between TGF-β1 and complement activation provides another insight into the pathogenesis of AS induced by complement activation.

Taken together, we demonstrated that the complement system was extensively activated in the spinal bone marrow in a PG-induced AS mouse model and in AS patients accompanied by the alteration of immune cells and cytokine especially TGF-β1, in which a bacteria-derived complement inhibitor, Efb-C, remarkably retarded the progression of mouse AS by inhibiting complement activation. Two important modulators, TGF-β1 and RANKL, involved in AS disease progression were elevated upon *in vitro* complement attack in osteoblast and/or osteoclast cells. Our findings establish a proof of concept that complement inhibition holds great potential for AS therapeutics.

## Materials and Methods

### Ethics Approval

Written informed consent was obtained from each patient and healthy control included in this study. The protocols used in human research and mouse study were approved by the Ethics Committee of Shanghai Changzheng Hospital affiliated to the Second Military Medical University and the Animal Ethics Committee at Shanghai Medical School, Fudan University, respectively. All related experiments were carried out in accordance with the approved guidelines.

### PG-induced AS mouse model

Susceptible BALB/c mice immunized with human cartilage PG may suffer from progressive polyarthritis which is frequently accompanied by spondylitis resembling human AS^41^. Therefore, we first prepared human cartilage PG according to the previous report^42^, and then immunized BALB/c mice to induce AS, as described previously^43^,^44^. Total 30 female BALB/c mice (24–26 week old, Slac Laboratory Animal Co., Shanghai, China) were divided into three groups (10/group) comprising of (1) Control group, intravenously injection of PBS alone, once per week; (2) PG group, intraperitoneally (i.p) injection of PG (100 μg/mouse) mixed with 200 μl adjuvant Dimethyldioctadecylammonium bromide (DDA, Sigma), once per two weeks for 7 times; and (3) PG + Efb-C group, PG/DDA immunization combined with intravenously injection of Efb-C (1 mg/mouse) dissolved in PBS, once per week for 14 times (If immunization with PG/DDA in a certain week, Efb-C injection was postponed for one day). At 14 weeks, the treated mice were anesthetized for osteoblast activity measurement and scarified to collect tissues and serum for future analysis.

### *In vivo* monitoring of osteoblast activity

Osteoblast activity was assessed *in vivo* using OsteoSense 750 EX probe, a bisphosphonate derivative labeled with NIR fluorescent agent (PerkinElmer, Waltham, MA). This compound binds to newly synthesized hydroxyapatite by osteoblasts, thus allowing visualization of areas of microcalcifications and bone remodeling. The compound (4 ng/100 μl/mouse) was injected intravenously (i.v), and the mice were imaged 24 hours later using IVIS Lumina III (PerkinElmer, Waltham, MA) with excitation filter of 749 nm and emission filter of 770 nm wavelength for 30 sec exposure time.

### Histology and immunohistochemistry

For histology, whole spine from control, PG, or PG + Efb-C treated mice were collected at experimental end-point, fixed overnight in 10% formalin, decalcified in 14% EDTA, embedded in paraffin blocks, cut into 5 μm sections, and stained with hematoxylin and eosin (H&E). Serial sections were assessed for evidence of inflammation, intervertebral disc and bone damage based on a previously described scoring system^44^.

For IHC staining, sections were incubated with anti-mouse C3b/iC3b/C3c (1:25; Hycult Biotech), rabbit anti-SC5b-9 neoantigen (1:50; Complement Technology, Inc.), and rat anti-mouse F4/80 (1:50; BioLegend Inc., San Diego, CA) at 4 °C overnight. Then detection of immunoreactivity was performed using a GTVision III immunohistochemical detection kit (GK500705; GeneTech, Shanghai, China) according to the manufacturer’s instructions. All sections were examined under light microscopy at x400 magnification and scored in a blinded fashion by two independent researchers according to previous studies with slightly modification^45^,^46^. Briefly, the entire slide was evaluated, and 5 fields were randomly chosen. First, a proportion score was assigned, which represented the estimated proportion of positively stained cells, in which the C3b/iC3b staining was only assessed in macrophages carefully (0, <5/100; 1, 5/100 to 33/100; 2, 34/100 to 66/100; 3, >66/100). Next, an intensity score was assigned, which represented the average intensity of positive cells (0, none; 1, weak, 2, intermediate; and 3, strong). The proportion and intensity scores were then added to obtain a total score, which ranged from 0 to 6. A score of 0 to 3 was considered negative, and a score of 4 to 6 was considered positive (4 scored as weak, 5 scored as intermediate, and 6 scored as strong).

In addition, due to the difficulties in obtaining biopsy at normal healthy axial skeleton, we only stained C3b/iC3b and MAC deposition in vertebrae facet joint tissues of two AS patients underwent orthopedic surgery without scoring.

### Flow cytometry analysis

For immune staining, spleens and bone marrow from mice were mechanically disaggregated to obtain single-cell suspensions. For removal of erythrocytes before staining, cell suspensions were treated with RBC Lysis Buffer (BioLegend Inc., San Diego, CA) on ice. Cells were then incubated with fluorochrome-conjugated mAbs to mouse F4/80 (APC), CD11b (FITC), NK-1.1 (PE), CD3ε (FITC), CD4 (FITC), CD25 (APC), FOXP3 (PE), CD8α (PE) and Gr-1 (APC) (BioLegend Inc., San Diego, CA) on ice for 30 min according to the manufacturer’s instructions. Samples were then assayed by a Cytomics FC500 MPL (Beckman Coulter, Inc., Brea, CA) and analyzed with FlowJo software (Tree Star, Ashland, OR).

### Multiplex Cytokine Analysis

The following cytokines and chemokines, TGF-β1, TGF-β2, TGF-β3, MIP-1α, IL-1α, IL-1β, IL-2, IL-3, IL-4, IL-5, IL-6, IL-10, IL-12(p40), IL-12(p70), IL-13, IL-17, Eotaxin, G-CSF, GM-CSF, IFN-γ, KC, MCP-1, VEGF, MIP-1β, RANTES, TNF-α, IL-18, FGF-basic and M-CSF were simultaneously measured in mouse serum *via* the TGF-β 3-plex Assay and Bio-Plex Pro™ Mouse Cytokine 23-plex Assay (Bio-Rad Laboratories, Inc., Hercules, CA) according to manufacturer’s instructions.

### Zymosan-induced complement activation *in vitro*

Briefly, 1 × 10^5^ RAW264.7 or MC3T3-E1 cells were suspended in 200 μl medium and plated in 6-well plates. For complement activation, cells were incubated with 25 μl pre-activated zymosan (Complement Technology, Inc., Tyler, TX) and 50% NHS as the source of complement or 50% IHS as control at 37 °C for 2 hours, then the reaction medium was replaced by normal culture medium. At the indicated time point, cell pallet or supernatant were collected for future analysis.

### Alizarin Red S staining

MC3T3-E1 cells (5 × 10^4^) were plated in 6-well plates. After 21 days differentiation induction using dexamethasone (10 nmol/L), phosphoglycerol (10 mmol/L), ascorbic acid-2-phosphate (0.5 mmol/L) with fresh medium replace twice per week, cell culture medium was aspirated and cells were washed with PBS for 3 times followed by fixation with 4% paraformaldehyde/PBS for 15 min at room temperature. Next, 1 ml Alizarin Red S Stain Solution (ScienCell Research Laboratories, Carlsbad, CA) was added to each well for incubation for 20–30 min according to the manufacturer’s instruction, and then the cells were washed with PBS 3 times for images collection under microscope. We further quantified the staining by desorbing Alizarin Red S in 10% cetylpyridinium chloride (solarbio science & technology, Beijing, China) for 1 hour. The supernatants were collected for absorbance reading at 570 nm in a spectrophotometer (Bio-Tek, Winooski, VT).

### *In vitro* osteoclastic differentiation and TRAP staining assay

RAW264.7 cells were cultured in 96-well plates at 3 × 10^3^ cells and treated with 50 ng/mL of recombinant murine RANKL (PeproTech Inc., Rocky Hill, NJ) as positive control or cell culture supernatant of MC3T3-E1 stimulated by zymosan-mediated complement activation. After 5 days, cells were fixed in 4% paraformaldehyde for 30 min and then stained for the osteoclast enzyme maker, tartrate-resistant acid phosphatase (TRAP) activity according to the manufacturer’s instructions (Sigma). In our study, TRAP-positive multinucleated cells (nuclei >3) were defined as osteoclasts using a light microscope.

### Statistical Analysis

Results are presented as means ± SEM. The significant differences were determined using one-way ANOVA when 3 groups were compared unless otherwise specified. Values of *p* < 0.05 were regarded as statistically significant. All statistical analyses were carried out using GraphPad Prism 5.0 (GraphPad Software, Inc., La Jolla, CA).

## Additional Information

**How to cite this article**: Yang, C. *et al*. Inhibition of Complement Retards Ankylosing Spondylitis Progression. *Sci. Rep.* **6**, 34643; doi: 10.1038/srep34643 (2016).

## Supplementary Material

Supplementary Information
