# Supplementary material for: Inhibition of Complement Retards Ankylosing Spondylitis Progression
Source: Sci Rep. 2016 Oct 4;6:34643. doi: 10.1038/srep34643 (PMC5048143; doi:10.1038/srep34643)
Supplement: Supplementary Information [file srep34643-s1.pdf]

Inhibition of Complement Retards Ankylosing Spondylitis Progression

Chaoqun Yang, Peipei Ding, Qingkai Wang, Long Zhang, Xin Zhang, Jianquan Zhao[1], Enjie Xu, Na Wang, Jianfeng Chen, Guang Yang, Weiguo Hu and Xuhui Zhou

ONLINE SUPPLEMENTARY MATERIALS AND METHODS

Preparation of recombinant Efb-C

We prepared the recombinant 6×His tagged Efb-C protein based on our previous study [1]. The expression and purity of Efb-C were determined by 15% SDS-PAGE. After removing endotoxin using removal resin kit (Houshiji Co., Xiamen, China), the endotoxin concentration of three batches was further measured by Tachypleus Amebocyte Lysate For Endotoxin Detection (Horseshoe Crab Reagent Manufactory Co.，Ltd.，Xiamen，China; Catalog No. CEG4405) to be as low as 0.08 ±0.02 EU/ml compared with 1.57 ±0.52 EU/ml before treatment. Then the protein was aliquoted and stored at -80℃ until use. The recombinant protein concentration was measured using the BCA assay.

Cell Culture

MC3T3-E1 (subclone 14) murine osteoblastic cells and RAW264.7 murine macrophages were obtained from the Type Culture Collection Cell Bank of Chinese Academy of Sciences, and cultured in α-MEM or DMEM medium supplemented with 10% (v/v) heat-inactivated fetal bovine serum (FBS) and 1% penicillin/streptomycin, respectively.

Complement-mediated hemolytic assay

The complement-mediated hemolytic assay was used to determine the efficacy of Efb-C on blocking classical complement activation as our previous report [2].

Real-time PCR

Total RNA was extracted from MC3T3-E1 or RAW264.7 cells using Trizol reagent (Invitrogen) according to the manufacturer's instructions. Then the Reverse Transcription System (Promega, Inc.) was used to synthesize cDNA from total RNA. Real-time PCR amplification was performed with SYBR Green Master Mix (Invitrogen) and gene-specific primers. The endogenous gene *β-actin* was regarded as an internal. All primer sequences are specified in online supplementary table S1.

Western blot analysis

Western blot was performed according to the standard process. Mammalian cell lysis/extraction reagent (Sigma-Aldrich, St. Louis, MO) was used to generate total cell lysates. Polyvinylidene difluoride (PVDF) membranes (Merck Millipore Inc., Billerica, MA) transferred with proteins were incubated with anti-RANKL antibody (1:2000;, Abcam Inc., Cambridge, MA) and subsequent secondary antibodies conjugated with horseradish peroxidase (Santa Cruz Biotechnology, Inc., Dallas, TX), then developed using SuperSignal West Femto Chemiluminescence Substrate (Thermo Scientific, Waltham, MA). The blots were visualized using an ImageQuant™ LAS 4000 biomolecular imager (GE Healthcare Bio-sciences).

ELISA

Antibody titers against Efb-C in PG-immunized mouse serum were determined by an ELISA assay. Briefly，the test was performed on 96-well microplates coated with recombinant Efb-C protein at 250ng/100μl/well. After incubation with serum samples at 37°C for 1 hour, the plate was washed by PBST 5 times. Then the goat anti-mouse secondary antibody conjugated with horseradish peroxidase was added. The color was developed using chromogenic substrate tetramethylbenzidine (TMB) and stopped at 10min using the stop solution, and read using a Synergy H4 Multi-Mode Microplate reader (Biotek, Inc., Winooski, VT) set to 450nm.

Serum samples from 27 patients of 22 AS, 5 PLID and 4 healthy controls were obtained and level of TGF-β1 was determined by Human TGF-β1 (ExCell Biology Inc., Shanghai, China) according to the manufacturer's instruction. The amount of mouse TGF-β1 in cell culture supernatants was determined by using mouse TGF-β1(ExCell Biology Inc., Shanghai, China) ELISA kits.

REFERENCES

1. Gao, Y.P., *et al.* A novel peptide can mimic extracellular fibrinogen-binding protein to block the activation of complement system. *Cell Biochem Biophys.* **66**, 753-757 (2013).

2. Hu, W., *et al.* A high-affinity inhibitor of human CD59 enhances complement-mediated virolysis of HIV-1: implications for treatment of HIV-1/AIDS. *J Immunol.* **184**, 359-368 (2010).

Supplemental Figures

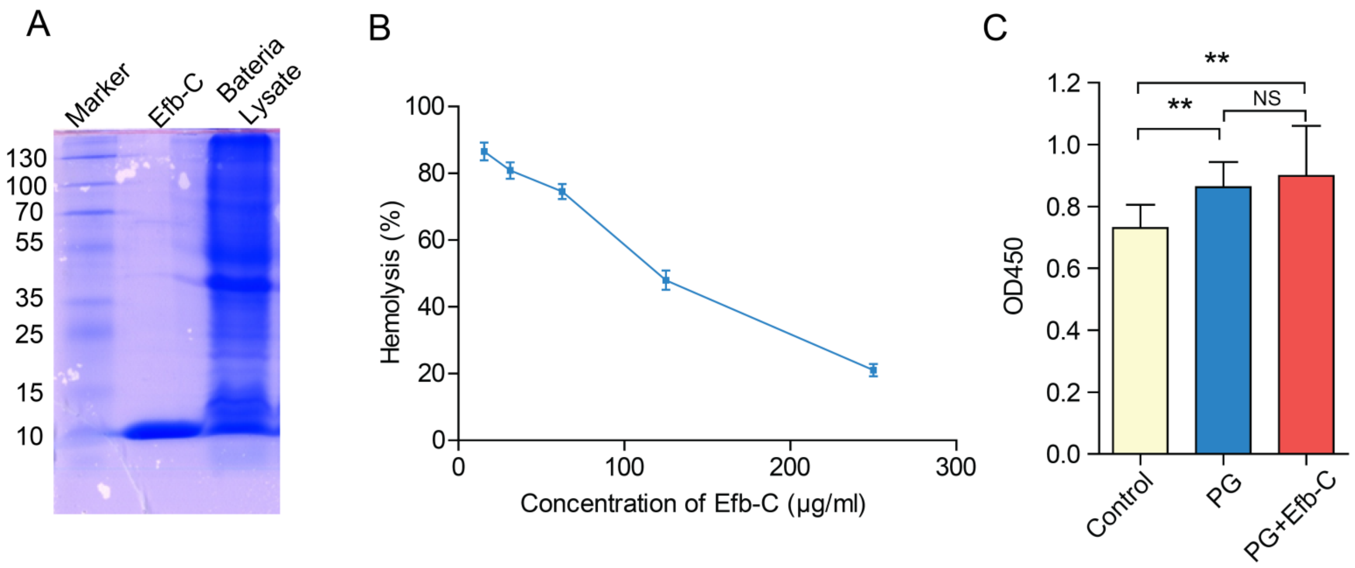

Figure S1. Preparation and characterization of Efb-C.

(**A**) Efb-C fusion protein tagged with 6×His was expressed in bacterial and purified by Ni-NTA (nickel-nitrilotriacetic acid) chromatography determined by 15% SDS-PAGE.

(**B**) Efb-C function for complement inhibition. Efb-C protects mouse erythrocytes from complement-mediated hemolysis. Mouse erythrocytes were pre-sensitized by rabbit anti-human erythrocytes polyclonal antibodies, then 25% NHS was added to activate complement in the presence of different concentrations of Efb-C.

(**C**) Efb-C immunogenicity. The titers of Efb-C antibody in sera of control, PG alone or PG + Efb-C treated mice were measured by ELISA. Efb-C combined with PG treatment did not increase the OD450 value compared with PG alone treatment, although both of their values were higher than that in control group. Data represent mean ± SEM. Control, n=10; PG, n=10; PG + Efb-C, n=9; **, $P < 0.01$.

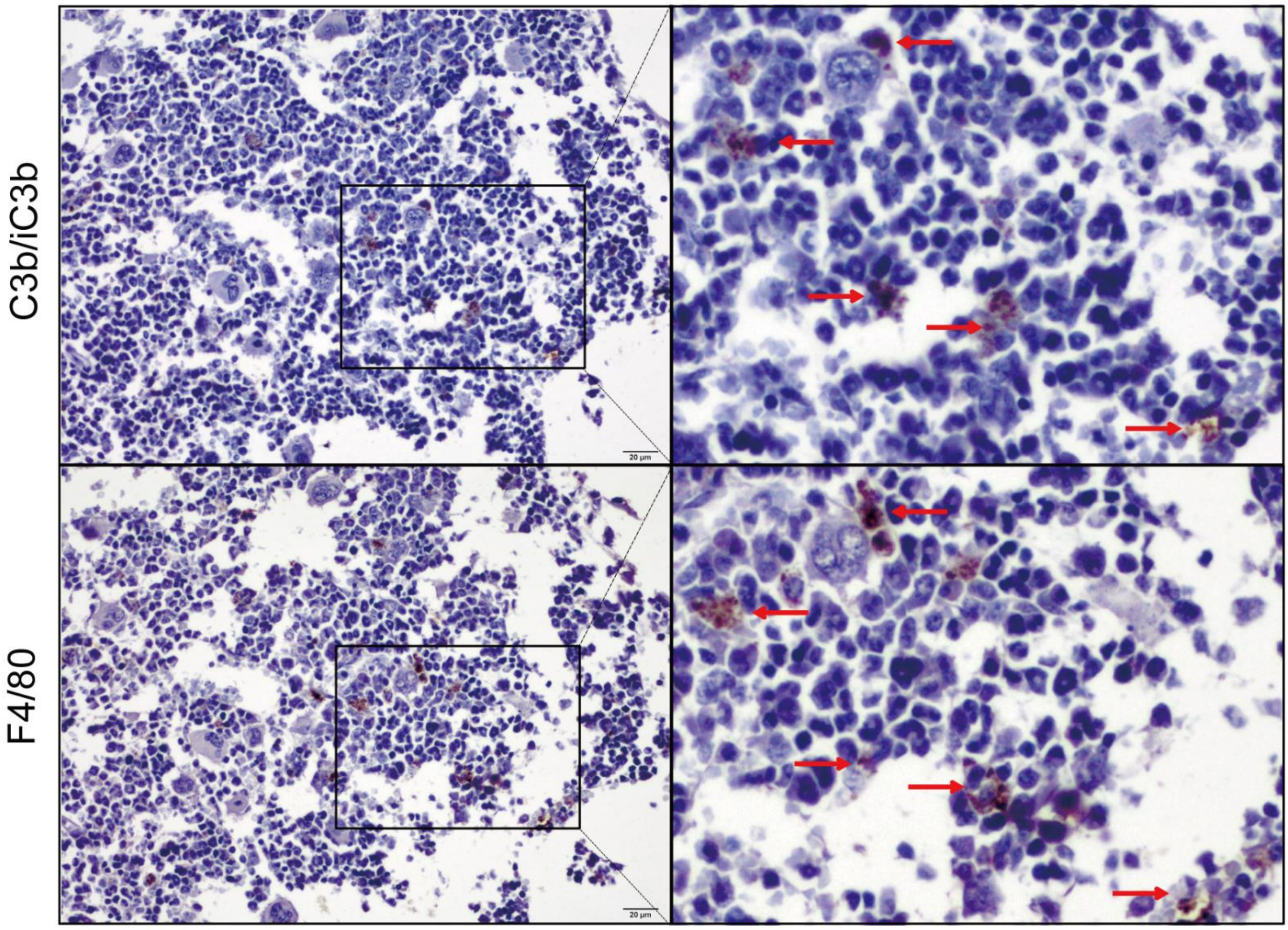

Figure S2. C3b/iC3b was exclusively deposited in macrophages of spinal bone marrow in PG-immunized mice.

IHC assay of serial sections determined the co-staining of C3b/iC3b and F4/80 in macrophages (red arrows). Scale bars, 20μm.

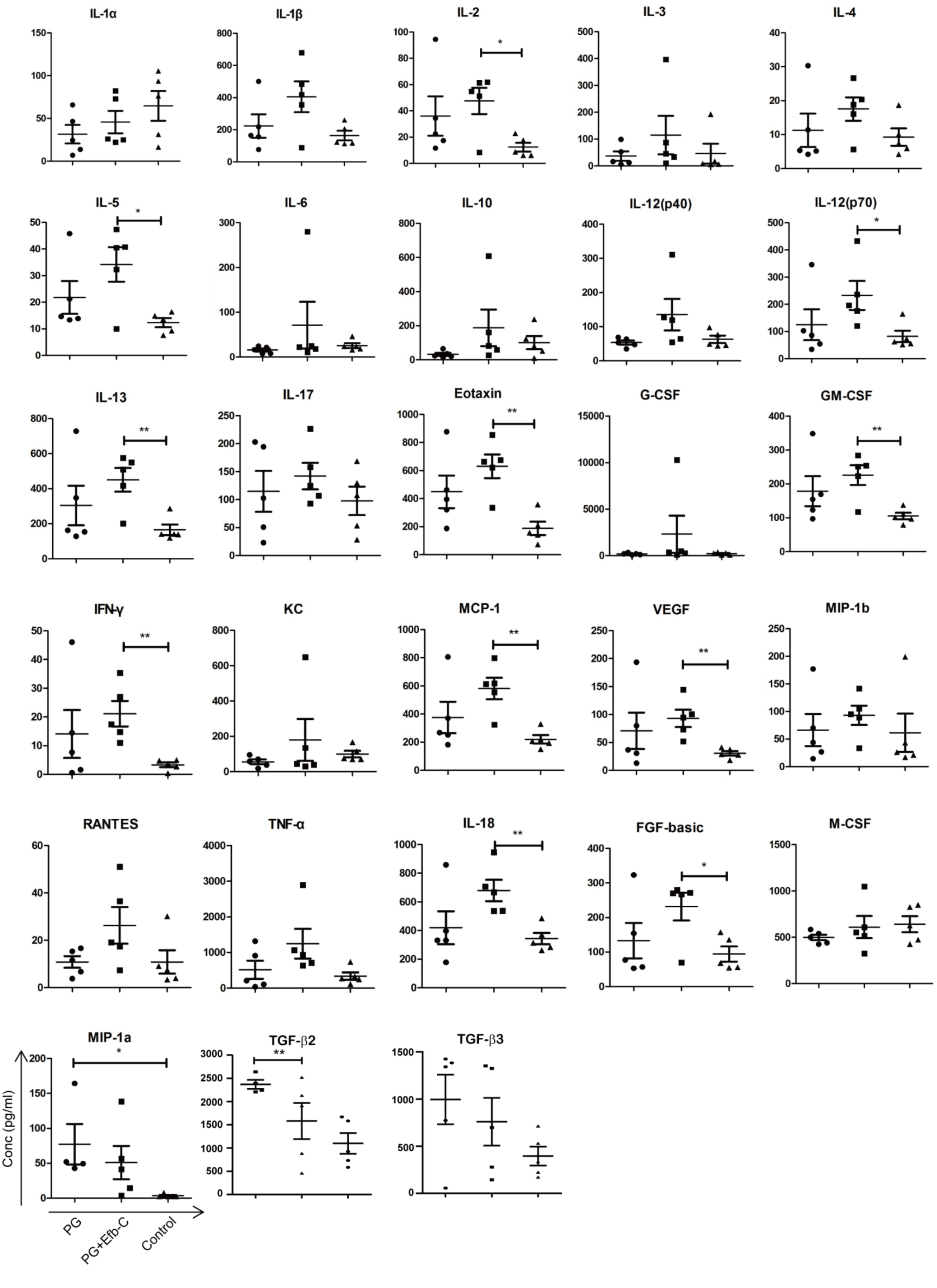
IL-1α
IL-1β
IL-2
IL-3
IL-4
IL-5
IL-6
IL-10
IL-12(p40)
IL-12(p70)
IL-13
IL-17
Eotaxin
G-CSF
GM-CSF
IFN-γ
KC
MCP-1
VEGF
MIP-1b
RANTES
TNF-α
IL-18
FGF-basic
M-CSF
MIP-1a
TGF-β2
TGF-β3
Conc (pg/ml)
PG
PG+Efb-C
Control

Figure S3. The expression of 28 cytokines in mouse sera in PG-induced AS model.

A Bio-Plex assay was employed to measure the concentration of a panel of cytokines in mouse sera. N=5 mice in each group, data represent mean ± SEM; *, $P < 0.05$; **, $P < 0.01$.

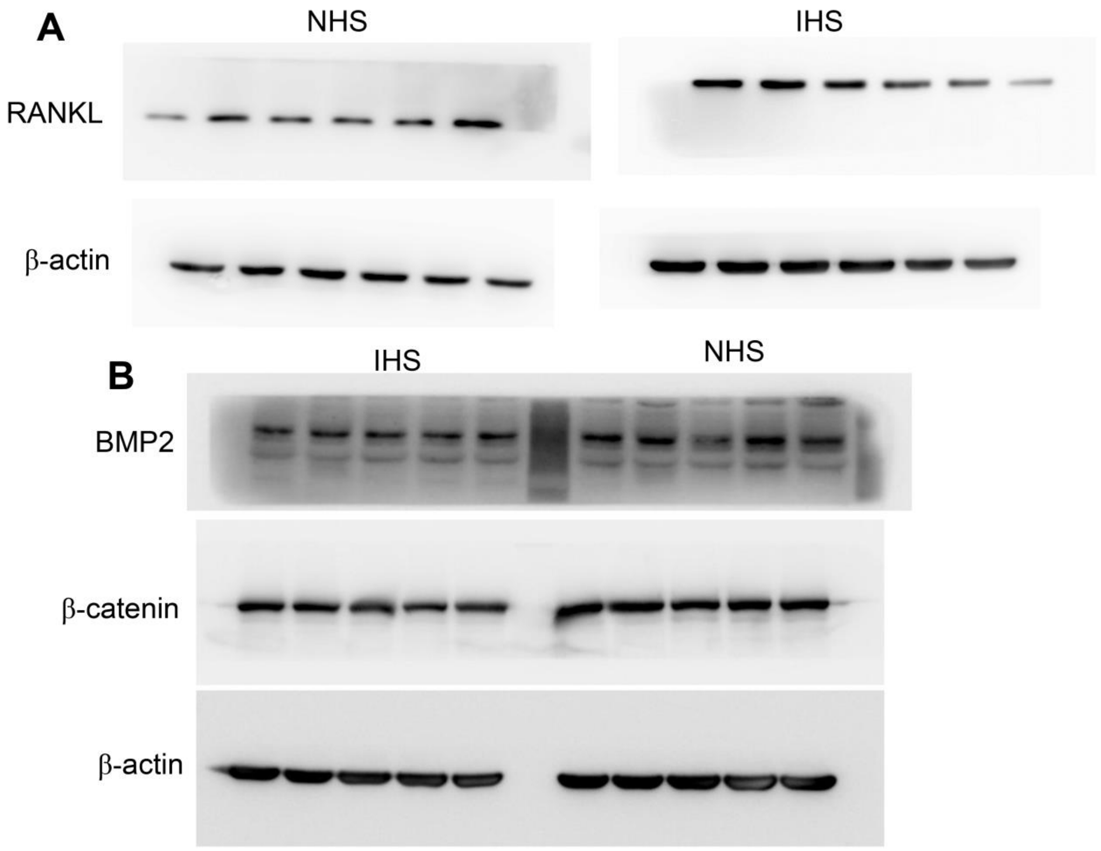

Figure S4. The original blot images corresponding with Figure 6D and E.

(**A**-**B**) Blot images corresponding with Figure 6D (A) and Figure 6E (B).

Supplemental Table 1. The primer sequences used in qRT-PCR.

Gene (NCBI ID)	Orientation	Sequence
Mouse *TGF-β1*	Forward	AGCTGCGCTTGCAGAGATTA
(NM_011577.1)	Reverse	AGCCCTGTATTCCGTCTCCT
Mouse *RANKL*	Forward	ACTGCCAGGACCTCTGTGAA
(BC137720.1)	Reverse	CGCAGGTACTTGCCGTAGTC
Mouse *β-actin*	Forward	TGGCTCCTAGCACCATGAAG
(NM_007393.4)	Reverse	CGCAGCTCAGTAACAGTCCG
